# Wind Turbine Main Bearing Fault Prognosis Based Solely on SCADA Data

**DOI:** 10.3390/s21062228

**Published:** 2021-03-23

**Authors:** Ángel Encalada-Dávila, Bryan Puruncajas, Christian Tutivén, Yolanda Vidal

**Affiliations:** 1 Mechatronics Engineering, Faculty of Mechanical Engineering and Production Science (FIMCP), Campus Gustavo Galindol, ESPOL Polytechnic University, Escuela Superior Politécnica del Litoral, ESPOL, Km. 30.5 Vía Perimetral, Guayaquil 090112, Ecuador; angaenca@espol.edu.ec (Á.E.-D.); bpurunca@espol.edu.ec (B.P.); cjtutive@espol.edu.ec (C.T.); 2 Control, Modeling, Identification and Applications (CoDAlab), Department of Mathematics, Escola d’Enginyeria de Barcelona Est (EEBE), Campus Diagonal-Besós (CDB), Universitat Politècnica de Catalunya (UPC), Eduard Maristany, 16, 08019 Barcelona, Spain; 3 Facultad de Ingenierías, Universidad ECOTEC, Km. 13.5 Vía a Samborondón, Guayaquil 092302, Ecuador; 4 Institute of Mathematics (IMTech), Universitat Politècnica de Catalunya (UPC), Pau Gargallo 14, 08028 Barcelona, Spain

**Keywords:** fault prognosis, wind turbine, main bearing, normality model, real SCADA data

## Abstract

As stated by the European Academy of Wind Energy (EAWE), the wind industry has identified main bearing failures as a critical issue in terms of increasing wind turbine reliability and availability. This is owing to major repairs with high replacement costs and long downtime periods associated with main bearing failures. Thus, the main bearing fault prognosis has become an economically relevant topic and is a technical challenge. In this work, a data-based methodology for fault prognosis is presented. The main contributions of this work are as follows: (i) Prognosis is achieved by using only supervisory control and data acquisition (SCADA) data, which is already available in all industrial-sized wind turbines; thus, no extra sensors that are designed for a specific purpose need to be installed. (ii) The proposed method only requires healthy data to be collected; thus, it can be applied to any wind farm even when no faulty data has been recorded. (iii) The proposed algorithm works under different and varying operating and environmental conditions. (iv) The validity and performance of the established methodology is demonstrated on a real underproduction wind farm consisting of 12 wind turbines. The obtained results show that advanced prognostic systems based solely on SCADA data can predict failures several months prior to their occurrence and allow wind turbine operators to plan their operations.

## 1. Introduction

Energy is a key pillar of human evolution. Currently, the challenge of obtaining energy while minimizing costs, and pollution is a matter of concern owing to climate change and global warming, as well as the need to democratize the extraction of energy worldwide. In this regard, renewable energy, i.e., energy collected from renewable resources, is an excellent option, as they are clean and exist over a wide geographical area, unlike fossil-fuel energy sources, which are air pollutants and are concentrated in a limited number of countries. Among renewable energy sources, the wind-energy sector has grown significantly in the last two decades. In 2019, wind energy generated enough electricity to meet 15% of Europe’s electricity demand [1] and was the leading source of new capacity in Europe, the U.S., and Canada, as well as the second largest in China. However, unleashing the massive potential of wind energy is crucial for reducing the levelized cost of electricity (LCOE) [2]. Increasing the size of wind turbines (WTs) and moving offshore, where steadier and higher wind speeds are available, are the two key factors in decreasing the LCOE. However, these two factors have accelerated the need for better condition monitoring strategies.

Condition monitoring is the process of monitoring a signal indicating the condition of machinery (vibration, temperature, etc.) to identify a deviation from the normal operation behavior, which is indicative of a developing fault. Condition monitoring is the crux of the matter to move from time-based preventive maintenance, which is still the current mainstream practice for WTs, to predictive maintenance, as it relies on the actual condition of the equipment rather than the average or expected life statistics. Because a failure is a process rather than an event, the earlier the process is detected, the more the flexibility that exists to manage it. Fault detection strategies usually warn about the appearance of a fault too late, and the fault is already mature when it is detected, which prevents proper planning of the maintenance operation [3,4,5]. Meanwhile, prognosis strategies provide a predictive maintenance option that gives the decision-maker the flexibility to decide whether and when to act before the subsystem or turbine fails. Thus, WT downtime is minimized, and the component lifetime is maximized. A significant amount of research on fault prognosis for WTs exists, some of which are reviewed in Reference [6]; the main subsystems that the majority of the research focus on include: blades (e.g., References [7,8]), gearboxes (e.g., References [9,10]), and bearings (e.g., References [11,12,13]). These aforementioned studies use data from specific and costly condition monitoring sensors, as they are mainly based on high-frequency vibration analysis, acoustic emission signals, or oil analysis sensors. In contrast, in this work, the proposed predictive maintenance strategy is achieved without needing to invest in additional hardware; it only requires the already available supervisory control and data acquisition (SCADA) data.

Although SCADA data have not been developed specifically for the purpose of condition monitoring, being able to extract relevant information from it could result in rapid deployment and modest set-up costs. SCADA data have been collected for long, but owing to the lack of appropriate data interpretation tools, they have not been considered for condition monitoring purposes. SCADA data are highly variable owing to the constantly changing operational conditions caused by the fluctuation of environmental conditions (such as wind speed and direction, turbulence intensity, ambient temperature, etc.), which are affected by seasonality. Furthermore, these parameters have a lower sampling rate (usually once every 10 min) compared to the kHz frequency of traditional condition monitoring strategies, they are rarely standardized, and the description of work orders is generally not clear. Thus, it is challenging for researchers to contextualize SCADA data for fault prognosis [14]. However, recent research has focused on this approach, and there are some success stories about using only SCADA data from real WTs for condition monitoring. It is important to note that using just SCADA data means that no extra sensors are used; however, some information from the SCADA alarm logs might be used but with limited fault detection and diagnosis, and no prognosis or functionality. Additionally, work orders could also be used for data labeling. For example, in Reference [15], the diagnosis and prediction of WT faults from SCADA data were accomplished using support vector machines (SVM), and, in Reference [16], a fault prognosis procedure was proposed using an a priori knowledge-based adaptive neuro-fuzzy inference system. By using a priori knowledge about faults (the data of six known WT pitch faults are used to train the system), the proposed system improves fault diagnosis. In Reference [17], a framework for automatically identifying periods of faulty operation using rules applied to the turbine alarm system are presented and applied to perform fault classification.

The aforementioned studies used SCADA data and validated their approach on real WTs; however, all of them required faulty data (historical fault data). Historical SCADA data must be accurately labeled with the periods when the turbines are down due to a fault, as well as with the cause of the fault. However, this is time-consuming, error-prone, and likely to result in a set of labeled vectors with an unbalanced number of classes. In contrast, in this work, there is no need for historical fault data; thus, the proposed strategy can be applied to any wind farm, even when no faulty data have been recorded. In this work, a normal behavior model is proposed, i.e., the model is built using normal (healthy) operation data. Heretofore, this introduction has focused on wind turbine fault diagnosis related references, but it is also important to note that analogous concepts to the ones proposed in this work are used in other areas of application. For example, error estimation and accuracy of machine learning methodologies have been performed on real datasets in different applications, e.g., Reference [18] (vessels). Likewise, the use of solely healthy data to diagnose non-previously occurred failures is used for cross-domain fault diagnosis problems in rotating machines, e.g., Reference [19].

This work deals with the main WT bearing faults. There are two main reasons for this selection. First, as stated by the European Academy of Wind Energy (EAWE) [20], the wind industry has identified main bearing failures as a critical issue in terms of increasing WT reliability and availability, as they lead to major repairs with high replacement costs and long downtime periods. Second, the authors had access to two years of SCADA data from a real underproduction wind farm (composed of 12 WTs), where main bearing failure occurred in one of the WTs; thus, it could be used to verify the performance of the proposed strategy in a real-life situation.

Most models in literature use simulated SCADA (e.g., Reference [21]) or experimental data but rarely real data. Furthermore, when dealing with real data, only one or two WTs are tested. For example, in Reference [22], an ensemble approach was proposed to detect anomalies and diagnose faults; however, it was only tested on two WTs and the warning was only given less than a week before failure, thus not leaving adequate time to plan the operation and maintenance action. In Reference [23], data from one WT in the South-East of Ireland were used to validate the proposed strategy; fault and alarm data were filtered and analyzed in conjunction with the power curve to identify periods of nominal and faulty operation. In contrast, this study used a wind farm composed of 12 WTs to test the proposed strategy.

The remainder of this paper is organized as follows: A brief description of the wind farm is provided in Section 2. The main bearing types and their faults are reviewed in Section 3. A description of the SCADA data is provided in Section 4. The proposed fault prognosis methodology is described in Section 5. The obtained results are presented and discussed in Section 6. Finally, the conclusions and future work are presented in Section 7.

## 2. Brief Wind Farm Description

**Figure 1:**
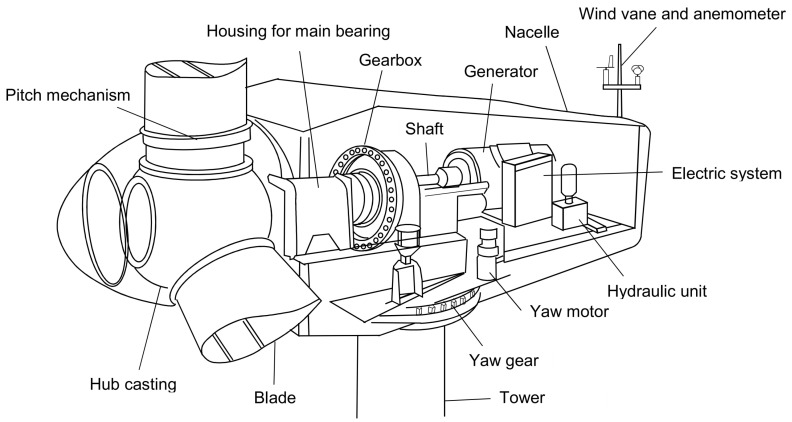
Main components of the wind turbine (WT) [24].

The wind farm is located in Spain and was commissioned in 2006. The WTs can generate 1500 kW of power and have a diameter of 77 m. Figure 1 shows the major components of these WTs. These are pitch-controlled WTs that not only use the pitch mechanism to brake, but also have an independent fail-safe piston accumulator on the blades. Additionally, they were equipped with a mechanical brake on a high-speed shaft. These turbines can also brake electromechanically using a generator to stop the rotation. Power production starts at a wind speed of 3.5 m/s. At 25 m/s, an automatic stopping occurs. The optimal performance can be achieved at a comparatively low wind speed of 11.1 m/s. This plant was certified according to IEC IIa. A summary of the technical specifications of the WTs is given in Table 1.

**Figure 2:**
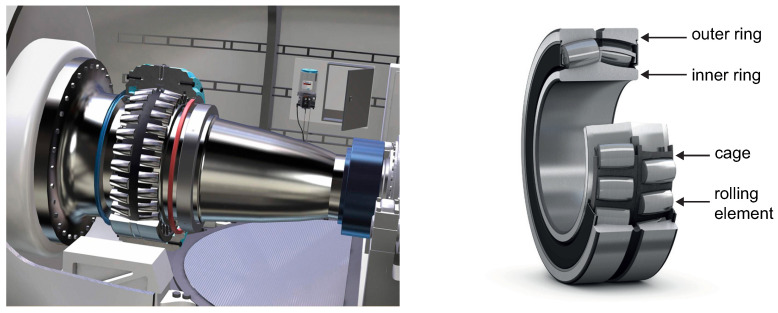
Spherical roller main bearing used in WTs. Courtesy of SKF.

It is noteworthy that these WTs use a double-spherical main roller bearing. These types of bearings are suitable for large radial loads and low to medium speeds, thus compensating for misalignment. Spherical roller bearings have two rows of symmetrical rollers, a common spherical outer ring raceway and two spherical inner ring raceways inclined at an angle toward the bearing axis. The center point of the sphere in the outer ring raceway is at the bearing axis. Figure 2 shows a spherical roller main bearing used in the WTs. As the main bearing is the component of interest in this work, a brief review on the main bearing types and their faults is given in the next section.

## 3. Main Bearing Faults

The main bearing is a large component inside a WT, and it can be damaged in a variety of ways. In this section, the main parts of this component and their different and various failure modes are discussed. The objective of this section is to show that there is no single pattern to predict a fault in this component, but rather a large number of possible patterns. This supports the idea of using unsupervised normality-based methods to predict the main bearing faults owing to the inability of supervised methods to predict failures outside their training dataset. However, should a supervised method be chosen, it would require a great variety of faulty data covering all failure types. Rolling bearings are composed of machine elements that permit the rotary motion of shafts for a wide range of applications, such as electric motors, aircraft gas turbines, gyroscopes, power transmissions, and WTs [25]. A typical rolling bearing consists of four elements: an inner race, an outer race, rolling elements that are in contact under heavy dynamic loads and relatively high speeds, and a cage around these rolling elements, as shown in Figure 2. Any of these parts are at risk of failure [26].

The SKF company classified the different failure modes of bearings by considering the ISO 15243 standard. This classification introduces the following failure modes [27]: (i) fatigue, (ii) wear, (iii) corrosion, (iv) electrical erosion, (v) plastic deformation, and (vi) fracture and cracking. These modes have different causes and behaviors, causing stress, deformation, micro-geometry destruction, cracking or spalling, shallow depressions, greyish-black patches, craters, and fractures. The objective of this work is to predict failures several months in advance, and, because all these failures develop progressively (i.e., develop through slow degradation), each one is briefly explained.

**Figure 3:**
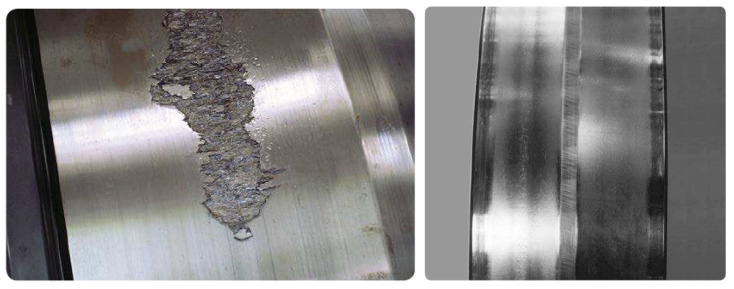
Fatigue failure. Subsurface-initiated (**left**) and surface-initiated (**right**) [27]. Courtesy of SKF.

First, there are two types of fatigue: subsurface-initiated fatigue and surface-initiated fatigue. Subsurface-initiated fatigue occurs beneath the contact surfaces of the raceways and rolling elements (see Figure 3, left). In contrast, surface-initiated fatigue occurs due to damage to the rolling contact surface asperities, which is generally caused by inadequate lubrication (see Figure 3, right).

**Figure 4:**
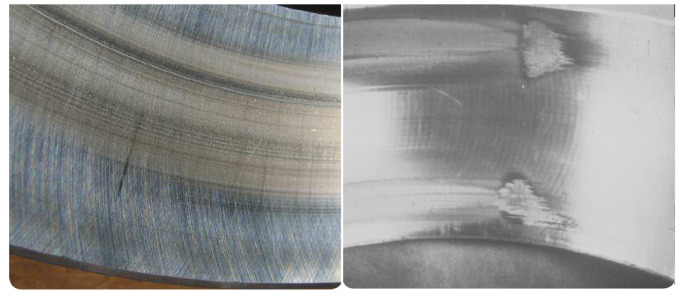
Wear failure. Abrasive wear (**left**) and adhesive wear (**right**) [27]. Courtesy of SKF.

Second, wear failure can be divided into two types: abrasive wear and adhesive wear. Abrasive wear is a degenerative process with the progressive removal of material, as shown in Figure 4 (left). In contrast, adhesive wear is a type of lubricant-related damage that occurs between two mating surfaces sliding relative to each other. It is characterized by the transfer of material from one surface to the other (called smearing). It is typically accompanied by frictional heat, which can sometimes temper or reharden the mating surfaces (see Figure 4, right).

**Figure 5:**
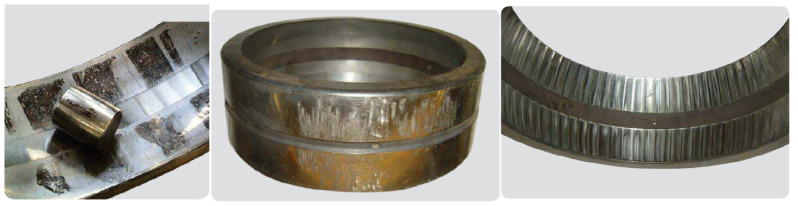
Corrosion failures. Moisture (**left**), fretting (**middle**), and brinelling (**right**) [27]. Courtesy of SKF.

Third, corrosion failures are divided into moisture corrosion, fretting corrosion, and false brinelling. Moisture corrosion occurs when a machine bearing is exposed to the ingress of water and other liquids as part of the operational process, resulting in greyish-black patches coinciding with the rolling element pitch, as shown in Figure 5 (left). Fretting corrosion occurs when there is relative movement between a bearing ring and its seat on a shaft or in a housing (see Figure 5, middle). Finally, false brinelling occurs in the contact area owing to the micromovements and/or resilience of the elastic contact under cyclic vibrations. The root cause is vibration during standstill (see Figure 5, right).

**Figure 6:**
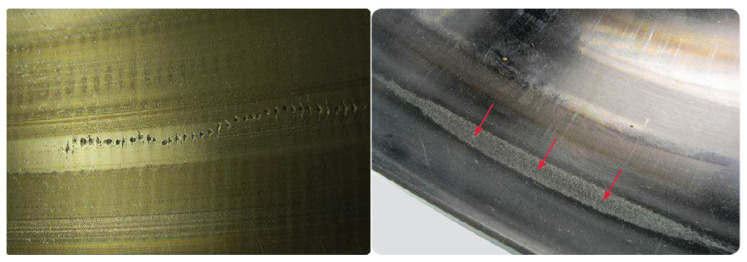
Electrical erosion failures. Excessive current (**left**) and current leakage (**right**) [27]. Courtesy of SKF.

Fourth, there are two types of electrical erosion: excessive current erosion and current leakage erosion. Excessive current erosion occurs when an electric current flows from one ring to the other via the rolling elements, causing damage. At the contact surfaces, the process is similar to that of electric arc welding (high current density over a small contact surface). The material is heated to temperatures ranging from tempering to melting levels, as shown in Figure 6 (left). However, in the initial stage of current-leakage erosion damage, the surface is typically damaged by shallow craters that are closely positioned and smaller in diameter compared to those from the damage from excessive current erosion (see Figure 6, right).

**Figure 7:**
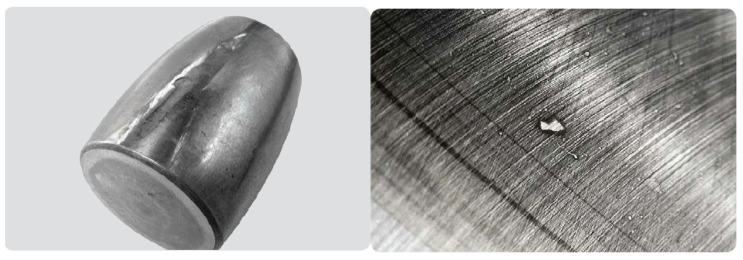
Plastic deformation failure. Overload (**left**) and indentation (**right**) [27]. Courtesy of SKF.

Fifth, plastic deformation can occur due to an overload or indentations from debris. Overload deformation can be caused by static overloading, shock loads, or improper handling, as shown in Figure 7 (left). In the case of indentations from the debris failure type, solid contaminants are introduced into a bearing via the seals or lubricant. They can also be the result of wear or damage to an adjacent component, such as a gear (see Figure 7, right).

**Figure 8:**
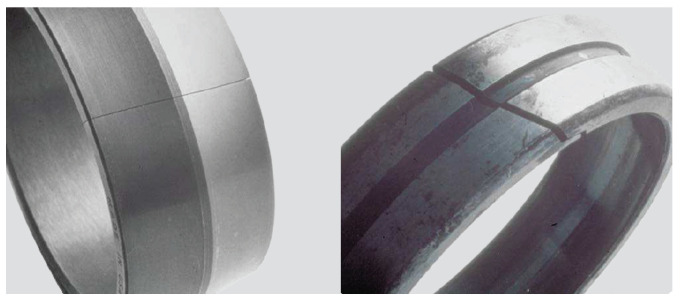
Fracture failure. Forced (**left**) and fatigue (**right**) [27]. Courtesy of SKF.

Finally, a bearing can be affected by forced fracture, fatigue fracture, or thermal cracking. A forced fracture occurs when the stress concentration exceeds the tensile strength of the material (see Figure 8, left). In contrast, a fatigue fracture starts when the fatigue strength of a material is exceeded under cyclic bending, as shown in Figure 8 (right). Finally, a thermal crack can occur when two surfaces slide against each other and generate frictional heat.

## 4. Real SCADA Data Description

The SCADA data used in this work were obtained from 12 operational WTs that can generate 1.5 MW. The continuous operational data were collected from the beginning of February 2017 till the end of November 2018. The wind farm SCADA datasets contain different variables that can be broadly grouped into environmental, electrical, component temperature, hydraulic, and control variables (see Table 2, Table 3, Table 4, Table 5 and Table 6). The mean, maximum, minimum, and standard deviation of the 10 min averaging period of 1 Hz sampled values are available for these variables.

Table 2 shows all the environmental related variables of the SCADA data, for example, ambient temperature, which affects the temperatures of all subsystems (the temperature of bearings changes significantly between winter and summer). It should be pointed out that the wind speed, which defines the different operating regions of the WT, is the most important exogenous variable related to the WT owing to its direct effect on the operation of the WT [28].

Table 3 shows the electrical related variables, such as the active power, which is sensitive to wind variations. The electrical energy is measured before it enters the distribution network to consider the consumption of the WT; therefore, it is considered as power delivered to the network. Electrical network frequency measurements and phase voltage measurements are also obtained to monitor possible fluctuations. Measurements of the power factor and reactive power are also collected to make adjustments, using capacitors, in the electrical system.

Table 4 shows the temperature related variables. As stated previously, this work focuses on the main bearing fault. Thus, it is important to note the low-speed shaft temperature, as this component is close to the main bearing, as well as the bearing temperatures at the coupling and non-coupling sides. In addition, note the variables related to the gearbox; the temperature readings were taken from the upper and lower gearbox radiators, and the lubrication of the gearbox was monitored using the hydraulic group oil temperature. Additionally, the generator temperature is another relevant variable.

The hydraulic variables obtained from the SCADA system are listed in Table 5. They include the pressure measurement of the general accumulator, the hydraulic group pressure, brake pressure, and general accumulated pressure of the blades. Each blade has an independent actuator with an accumulator to position the blade according to the mode of operation.

Table 6 shows the control related variables. The WTs are equipped with blade pitch control, which adjusts the blade’s angle of inclination to control the rotor speed and can execute the rotor brake in the feathered position. Another important control system is the yaw controller, which ensures that the nacelle is oriented correctly. Additionally, the rotor and generator speeds are crucial variables to control the WT operation.

Note that, apart from the SCADA data, information regarding maintenance and repair actions (work orders) were also available. This data provided information on the failures that occurred when they occurred, when the work was carried out, and information about the subsystem that was repaired or replaced. From this information, WT number 2 in the wind farm (WT2, from now on) had a main bearing fault on 21 May 2018. This information was used in this work to test whether the proposed methodology is capable of predicting the appearance of this fault months in advance.

## 5. Fault Prognosis Methodology

In this section, the proposed methodology is comprehensively described. First, the data preprocessing, which is performed to deal with real data that contains outliers and missing data, is thoroughly explained in Section 5.1. Second, the data split into training and test sets is given in Section 5.2. In this section, emphasis is placed on why the usual strategy of data shuffle must never be used in the context of time-series data. It will also explain how it is ensured that the anomalies detected are not just a change in seasonality. Furthermore, it is urged that almost one whole year of data is used for the training set. Then, in Section 5.3, the normality model for each WT is constructed based on an ANN. The ANN was trained to obtain a model that, from the input variables (at different time steps), can estimate the value of the main shaft temperature (at a specific time step), as a virtual sensor, when healthy data are provided. Fourth, in Section 5.4, the specific details of the ANN architecture are provided. Fifth, in Section 5.5, the application of Bayesian regularization, where the weights are regularized to improves the generalization without requiring a validation set, is discussed. Sixth, Section 5.6 discusses how to ensure that the data used to construct the normality model are healthy. Finally, a fault prognosis indicator is introduced in Section 5.7 to minimize the number of false positives (false alarms).

### 5.1. Data Preprocess

**Figure 9:**
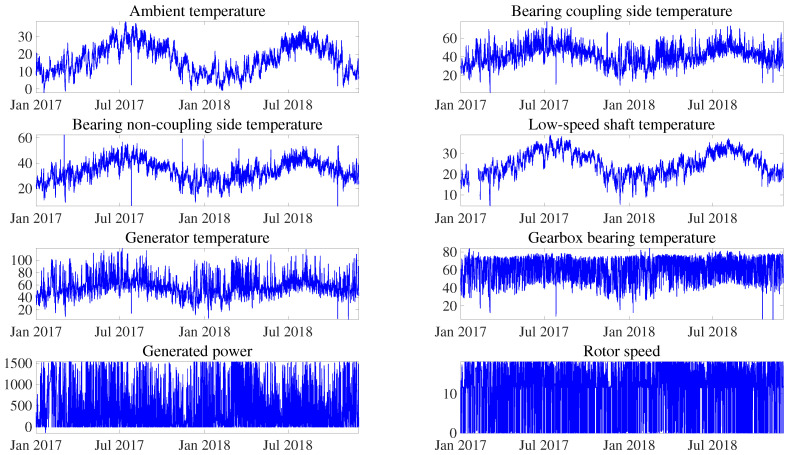
Plot example of the selected supervisory control and data acquisition (SCADA) variables used to develop the normality model. All of them are related to the mean value over a 10-min period.

In Section 3, the diverse main bearing failure modes are described, stating that, in many cases, they lead to an increase in temperature. For this reason, to build the normality model, the temperatures of the components located close to the main bearing are selected together with the ambient temperature, as it affects the temperatures of all subsystems. Additionally, the generated power and rotor speed provide information about the region of operation of the WT. The selected variables are shown in Figure 9 and are detailed in Table 7. Note that these variables are filtered through a range of realistic values for each sensor. Table 7 lists the specific ranges used for each sensor.

**Figure 10:**
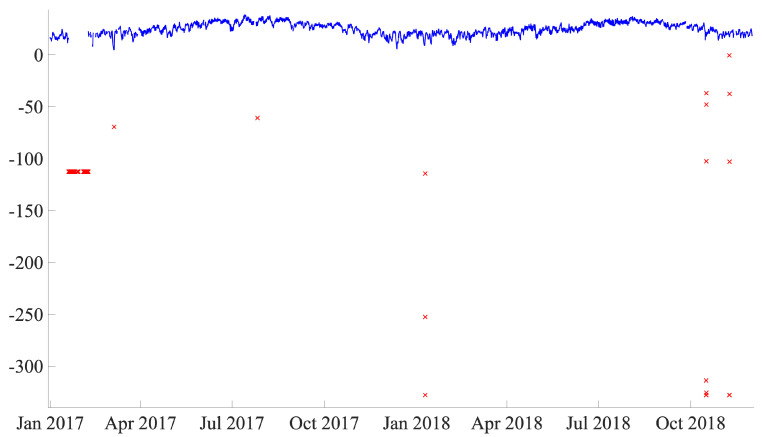
Out-of-range values are detected as outliers (red crosses) and assigned as missing values from the raw signal.

Data cleaning is an important step prior to the application of data analysis because noisy data is removed that could cause interference in the study. Furthermore, when the study is based on real data, missing data and outliers are unavoidable (unlike when the work is based on simulated/synthetic data). In this work, extreme values (outliers) are not systematically removed because, as stated in Reference [29], it could lead to loss of information related to fault prediction. Conversely, the use of manually defined ranges based on realistic values that can be obtained by different sensors could be a better strategy. In this work, out-of-range values are first set as a missing value and then filled using the same strategy as that used for the original missing values. Figure 10 shows an example of the values outside the range for the low-speed shaft temperature.

**Figure 11:**
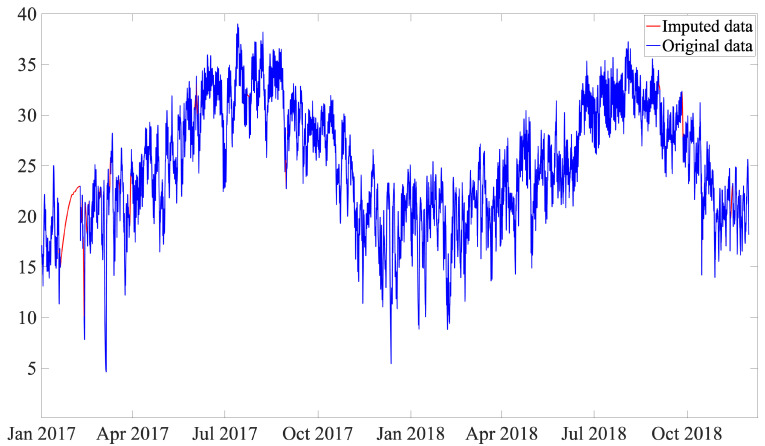

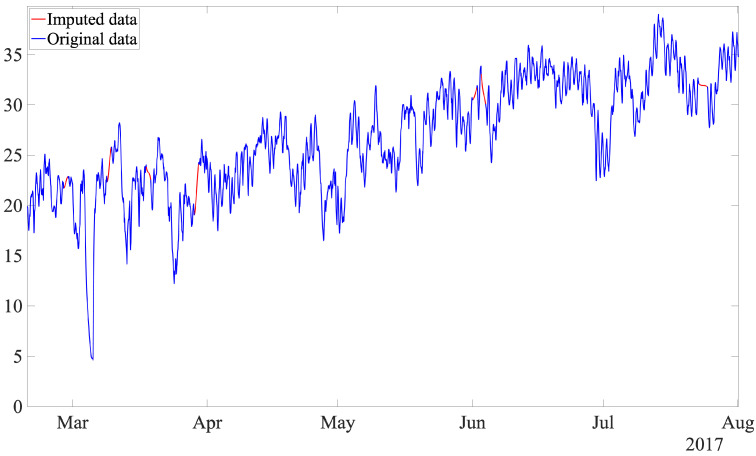
Low-speed shaft temperature raw data (without outliers) versus imputed data (**top**) and zoom in of the imputed data (**bottom**).

As out-of-range values are removed, the number of missing values is increased; thus, there is a need for a data imputation strategy. Imputations with mean, median, and mode are simple techniques; however, this can introduce a bias in the mean and deviation [30]. In this work, a single imputation, while avoiding complex mathematical calculations, is proposed by using the piecewise cubic Hermite interpolating polynomial (pchip) [31]. This polynomial works for the given data points with specified slopes at the interpolation points. A meaningful property of this strategy is that the obtained polynomial preserves the shape of the data and respects monotonicity, and guarantees that at least the first derivative is continuous. This interpolation strategy has been used in a variety of applications, such as calculating the signal-to-noise ratio in scanning electron microscopy (SEM) images [32], sampling smoothly predefined kinematic grids in high-energy particle collision problems [33], and decomposing nonlinear and non-stationary electromagnetic interference signals [34]. Figure 11 shows the original and imputed data, where the polynomial is computed and traced between the data points, considering the shape and continuity of the curve. Note that, for missing values that are at the beginning or at the end of the dataset, the closest value after or before the missing values is used.

Finally, data from selected variables have different sources; therefore, the values have different orders of magnitude. Thus, it is highly recommended to scale the data prior to use in any machine learning approach. In this work, max-min scaling is selected to scale the data. Considering that the Bayesian regularization algorithm is used (see Section 5.5), the best results are obtained if the training data are first mapped into the range [−1, 1] (or some similar region) [35]. Thus, max-min normalization was selected instead of the standard Z-score. Max-min normalization guarantees the data into the range [0, 1]. It is a simple technique, and its only disadvantage is coping with outliers, which has already been solved by using range filtering of the data.

### 5.2. Data Split: Train and Test Sets

The basic steps in all neural network based models are: (1) divide the data into training and test datasets, (2) use the training dataset to train the neural network, and (3) evaluate the model using the test dataset to determine how well it predicts (generalizes). In this section, we focus on the first step.

How available data are split into training and test sets plays a fundamental role in the construction of ANNs and has a significant impact on the obtained model. In this work, a fault prognosis methodology that is insensitive to both operating and environmental conditions is sought; therefore, the training and test datasets must have data from all the working conditions.

**Figure 12:**
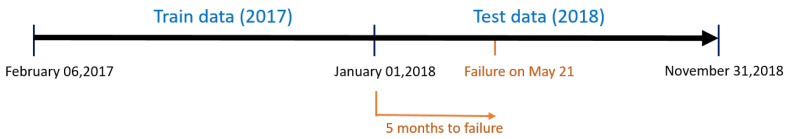
WT2 (WT number 2 in the wind farm) data for training and test.

It is noteworthy that, in this work, the training and test datasets have not been shuffled, as this can cause data leakage owing to the presence of strong time-series effects in the data [36]. Consequently, the training and test datasets were split in such a way that each set had one year of data. This approach ensures that the detected anomalies are not due to seasonality [37], and the model can cope with various operating and environmental conditions. Therefore, the available SCADA data were divided as follows: data corresponding to 2017 (47,232 samples) were used for training, and data from 2018 (43,920 samples) were used for testing. This data split was carried out for the entire wind farm. For example, Figure 12 shows the training and test data associated with WT2 (which had a main bearing fault on 21 May 2018).

As can be seen, in this work, there is no validation set because Bayesian regularization is used to train the ANN, as shown in Section 5.5.

### 5.3. Normality Model Based on an Artificial Neural Network (ANN)

**Figure 13:**
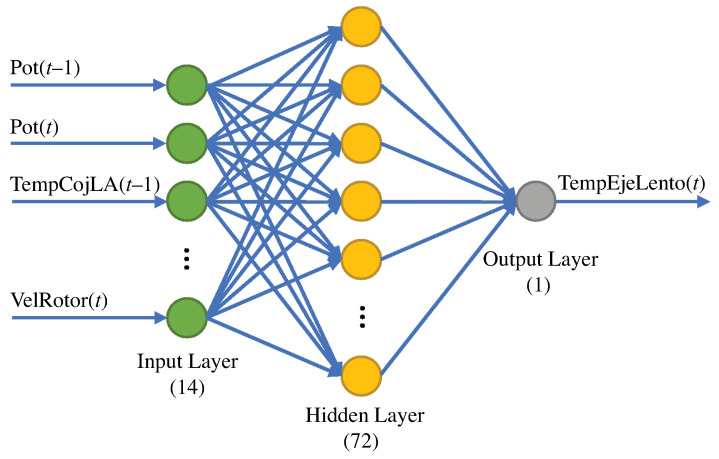
ANN model with 14 inputs, 72 neurons in the hidden layer, and 1 output.

The ANN model structure is proposed in this section and is based on the eight selected variables shown in Table 7. The output of the ANN is considered to be the temperature of the low-speed shaft (variable of interest) at time *t*, and the inputs are the remaining seven variables shown in Table 7 at time t−1 and *t*. Thus, referring to the structure of the ANN, there are 14 inputs and 1 output with a hidden layer comprising 72 neurons. Figure 13 shows the ANN structure.

The next sections provide a detailed explanation of the optimization method, the regularization method, and the selection of the number of neurons in the hidden layer.

### 5.4. Setup of the Proposed ANN

To provide a comprehensive reasoning of the hyper-parameter setup of the proposed ANN, a brief review of the Levenberg-Marquardt (LM) optimization method is given here to introduce the notation used.

First, note that the problem to be solved is

$$\underset{\beta }{\mathrm{argmin}}F\left(\beta \right)=\frac{1}{n}\sum_{i=1}^{n}{\left(T_{i}-{\hat{T}}_{i}\left(\beta \right)\right)}^{2},$$

where β is the vector of parameters (in this work, it includes the weights and biases of the ANN, i.e., β=(w,b)), *n* is the total number of samples in the training dataset, $T_{i}$ is the temperature value of the low-speed shaft given by the SCADA data for sample *i*, and ${\hat{T}}_{i}$ is its estimation given by the output of the ANN. In other words, the problem consists of minimizing the mean squared error, which can be rewritten as

$$\underset{\beta }{\mathrm{argmin}}F\left(\beta \right)=\frac{1}{n}\sum_{i=1}^{n}r_{i}{\left(\beta \right)}^{2},$$

where $r_{i}\left(\beta \right)=T_{i}-{\hat{T}}_{i}\left(\beta \right)$ are the residuals. Numerical optimization algorithms are usually used to address this problem. They are iterative procedures that update the parameters at each iteration as follows:

$$\beta_{k+1}=\beta_{k}+\delta_{k},$$

where *k* is related to the *k*th iteration of the algorithm, and $\delta_{k}$ is the increment to be determined by the specifically selected algorithm. The gradient descent (GD) algorithm adopted the following increment:

$$\delta_{k}^{GD}=-\mu J^{T}r\left(\beta_{k}\right),$$

where μ is the learning rate, $J^{T}$ is the Jacobian matrix transpose of the objective function *F*, and *r* is a column vector containing the residuals at each sample, that is, $r={\left(r_{1},r_{2},\cdots ,r_{n}\right)}^{T}$. In contrast, the Gaussian Newton (GN) algorithm uses as an increment:

$$\delta_{k}^{GN}=-H^{-1}J^{T}r\left(\beta_{k}\right),$$

where *H* is the Hessian matrix of the objective function *F*. Alternatively, the LM algorithm applies an increment:

$$\delta_{k}^{LM}=-{\left(H+\mu I\right)}^{-1}J^{T}r\left(\beta_{k}\right),$$

where *I* is the identity matrix, and μ is a nonnegative scalar parameter, usually called the damping parameter. Note that, when μ=0, the Gauss-Newton method is obtained, and, when μ is large, the method resembles gradient descent [38,39]. The fundamental idea behind the LM algorithm is to accomplish a performance similar to gradient descent when far away from the optimum, and to attain a performance similar to the Gauss-Newton method when close to the optimal value (to achieve fast convergence when being at the minimum neighborhood). To obtain this behavior, the damping parameter μ is adjusted at each iteration of the algorithm. In this work, it is raised by a factor of 10 if the current step fails to reduce the objective function, and it is decreased by a factor of 0.1 otherwise. The assigned initial value was μ=0.005. In this work, the LM algorithm terminates when at least one of the following conditions are met:

- A maximum number of 1000 epochs (using mini batches of size 128) is reached.
- The magnitude of the gradient, $J^{T}r\left(\beta_{k}\right)$, drops below the threshold $\varepsilon =10^{-7}$.
- The damping parameter, μ, exceeds its maximum possible value that has been set to $10^{10}$.

Note that the network used rectified linear unit (ReLU) activation functions, and initialization was performed using the Xavier initializer. Finally, to prevent overfitting, L2 regularization was introduced into the neural network using Bayesian regularization. Details are provided in the next section.

### 5.5. Bayesian Regularization

In this study, Bayesian regularization was employed to train the ANN [35]. This regularization can be applied to multi-layer feed forward ANNs that are used for nonlinear regression, which is the case at hand. MacKay [40] comprehensively contributed to the utilization of Bayes’ rule for NN training and regularization. First, in the Bayesian scheme, the ANN weights are considered as random variables, and their density functions are updated according to Bayes’ rule. Second, the training aims to minimize the objective function. Recall that, in this study, the mean squared error is minimized:

$$E\left(w,b\right)=\frac{1}{n}\sum_{i=1}^{n}{\left(T_{i}-{\hat{T}}_{i}\left(w,b\right)\right)}^{2},$$

where *w* and *b* are the parameters (weights and biases, respectively) of the ANN. When L2-regularization is used, an additional term is added to the objective function [41].

$$E_{R}\left(w\right)=\sum_{j=1}^{m}w_{j}^{2},$$

where *m* is the total number of weights in the ANN. Thus, the objective function *F* becomes:

$$F\left(w,b\right)=\alpha E\left(w,b\right)+\lambda E_{R}\left(w\right),$$

where α and λ are parameters whose relative values rule the priority for training and/or regularization, respectively. For instance, when α>>λ, the training optimization algorithm minimizes errors on the training dataset, but it may lead to overfitting. However, when α<<λ, the training optimization algorithm will give priority to weight size curtailment (in exchange for some errors in the training dataset), thereby generating a smoother model. The main challenge in adding regularization is to set proper values for these parameters. To handle this problem, Bayesian regularization considers the application of Bayes’ rule to optimize their values at each iteration of the numerical optimization [35]. A disadvantage is that this optimization requires the computation of the Hessian matrix of the objective function *F*. However, this can be approximated using the Gauss-Newton approximation, which, as noted in Reference [35], is readily available when the LM optimization algorithm is used [42] for training. A brief review of this method is given in Section 5.4 to introduce the notation used and thoroughly describe the hyper-parameter setup of the proposed ANN.

One benefit of Bayesian regularization is that it provides the so-called effective number of parameters, γ, which is a measure of how many network parameters (weights and biases) are effectively used to reduce the objective function [35,40]. If the final effective number of parameters is very close to the actual total number of parameters in the network, then the neural network may not be sufficiently large. In this case, more hidden layer neurons should be added and retrained; however, if the larger network has the same final γ value, then the smaller network is sufficiently large. Otherwise, more hidden layer neurons may need to be added. Finally, when a sufficiently large network has been trained for a sufficient number of iterations to ensure convergence, γ remains approximately the same, regardless of the total number of parameters in the network. That is, if an even larger network was tried, the network response would never overfit the data. This greatly simplifies the hyper-parameter tuning required to determine the optimum network size. In this study, as shown in greater detail in Section 6, a value of γ=1058 was obtained from a total of 1153 parameters in the proposed network (number of weights and biases).

For the sake of completeness, note that the formulas to compute at each iteration, *k*, the effective number of parameters, γ, and the objective function parameters α and λ are as follows [35]:

$$\gamma_{k+1}=n-2\alpha_{k}tr{\left(H\right)}^{-1},\;\alpha_{k+1}=\frac{n-\gamma_{k+1}}{2E(w,b)},\;\lambda_{k+1}=\frac{\gamma_{k+1}}{2E_{R}\left(w\right)},$$

where tr(·) stands for the trace operator.

Finally, note that Bayesian regularization regularizes the weights and improves the generalization of the neural network; thus, a validation set is not required (as its main purpose is to accomplish regularization and generalization of the model).

In summary, in this work, the LM optimization algorithm was used for training optimization, and Bayesian regularization was introduced to regularize the weights and improve the generalization of the constructed model without requiring a validation set.

### 5.6. Discussion on How to Ensure That Data Used to Construct Normality Model Is Healthy

On the one hand, the proposed normal behavior model relies on the fact that healthy data are used to train it. From a pure definition point of view, this model is not completely unsupervised but is semi-supervised, as it is required to ensure that the training data are healthy. On the other hand, having complete assurance that the data used to train is healthy is not an easy task, as the absence of work orders does not guarantee that the data is completely healthy.

At the studied wind farm, there is an extra wind turbine, WT13, which is not included in the results of our methodology as it had a fault during 2017 (year used for training). Thus, it is not possible to construct a normality (healthy) model for a WT, as there are faulty data during the period decided to be used as training. However, in this section, we show the training error output when a model is built using this WT. The result is a huge training error of 30.4969, which is clearly inadmissible. Table 8 shows the training error for the rest of the WTs in the park that have healthy data during the training period (2017).

In summary, the final train mean square error provides an estimate of the validity of the model. That is, when a WT model is harder to obtain (a much higher train mean square error is obtained), this could be a signal that the data has some anomalous behavior (it is not healthy) even if no work orders are reported. In this case, it is highly recommended to double check whether this turbine had issues during the year used as training.

### 5.7. Fault Prognosis Indicator

Typically, fault-detection indicators are defined using residuals and establishing a detection threshold. When a sample has a residual higher than the detection threshold, an alarm is triggered. However, in this case, if the residual, $|T-\hat{T}|$, was used directly to establish a threshold above which it is decided to give the alarm signal, this would lead to a non-assumable number of false positives (false alarms) that would render the method useless. This fact is further explained in the results section. However, in this section, an indicator to overcome this problem is introduced.

As already mentioned, if the indicator is based on the residual of a single sample, there would be an excessive number of false alarms. Thus, it is important to define an indicator that considers the persistence of consecutive samples above a specified threshold. In particular, a threshold was first defined based only on the training data residuals. The mean μ and standard deviation σ of the residuals over all training data are computed. Then, the threshold is defined as follows:

(1) threshold=μ+6σ.

Next, for each week in the test dataset, the number of samples that had a residual value greater than the threshold was counted and denoted as $n_{over}$. It is desired that the indicator has a range between 0 and 1. Thus, a minimum function is used to define the indicator in such a way that its value is 0 when no sample exceeds the threshold in that week, and a value of 1 when 504 samples (remembering that a week has 1008 samples) or more exceed the threshold in that week. Thus, the implemented weekly indicator is given as follows:

(2) $$\mathrm{indicator}=\min\left(1,\frac{n_{over}}{504}\right).$$

## 6. Results on a Real Wind Farm

The results of the proposed fault prognosis methodology for the entire wind farm is presented and discussed in this section.

**Figure 14:**
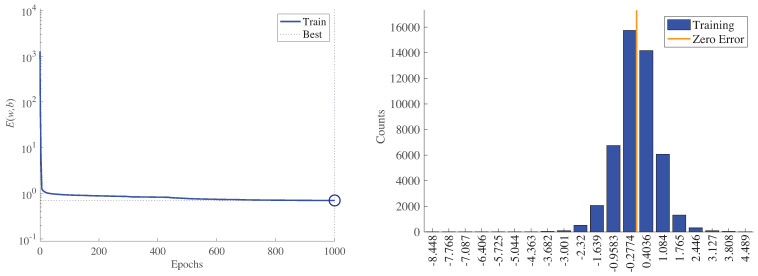
Minimization of the MSE, E(w,b), during training of WT1 (**left**). Error histogram with 20 bins of final training error over all training samples for WT1 (**right**).

**Figure 15:**
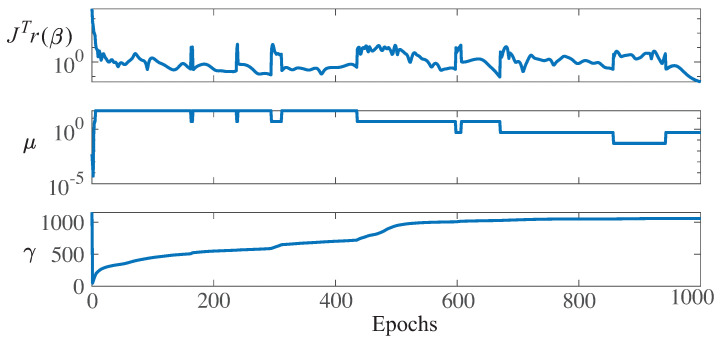
Values at each training epoch iteration for the gradient, $J^{T}r\left(\beta \right)$, damping parameter, μ, and effective number of parameters, γ, for WT1.

First, Figure 14 (left) shows the minimization of the MSE, E(w,b), during training for WT1. The best performance was 0.6984, and it is reached at the last epoch (1000). Recall that the target value is the low-speed shaft temperature; thus, the MSE has a direct physical interpretation in degrees Celsius. In addition, Figure 14 (right) shows a histogram (with 20 bins) of the final training error over all training samples for WT1. Note that the four bins with more counts have an error smaller than or equal to one degree Celsius. Furthermore, Figure 15 shows the parameter values related to the LM optimization algorithm and Bayesian regularization for WT1 training. Note that at epoch 1000, the following values were obtained: The gradients were $J^{T}r\left(\beta_{1000}\right)=0.0421$, the damping parameter was μ=0.5, and the final number of effective parameters used by the ANN was γ=1058. A larger network with an increased number of neurons in the hidden layer led to the same number for the parameter γ, thus proving that the size of the presented ANN (with 72 neurons in the hidden layer) was large enough.

Next, recall that Table 8 in Section 5.6 shows the final training error for each WT in the park. There is some variability among the different wind turbines, with minimum and maximum values of 0.6984 and 6.1074, respectively. These are acceptable values, related to the real-life variability among WTs due to, for example, different locations in the park. However, it is important to note that, when the values of the training error are much higher, then it should be considered that the training data might not be completely healthy. Most likely, if the ANN was not capable of significantly reducing the MSE for that specific WT, it is because the training data have some kind of anomaly. Recall that our main purpose is to construct a normality (healthy) model; thus, it is essential to have at disposal normal (healthy) training data.

**Figure 16:**
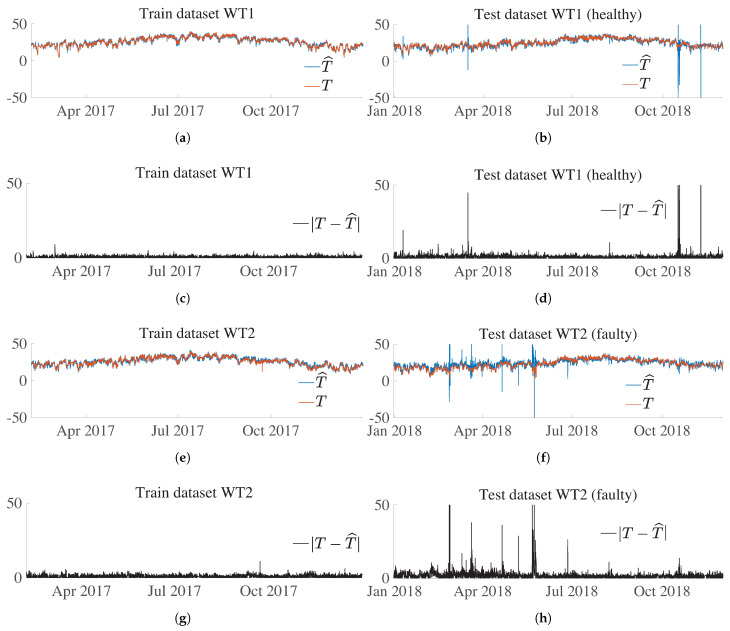
(**a**) ANN predicted value ($\hat{T}$) and target (*T*) value for WT1 over the train dataset. (**b**) ANN predicted value ($\hat{T}$) and target (*T*) value for WT1 over the test dataset. (**c**) Absolute difference value between the prediction and estimation, $|T-\hat{T}|$, for WT1 over the train dataset. (**d**) Absolute difference value between the prediction and estimation, $|T-\hat{T}|$, for WT1 over the test dataset. (**e**) ANN predicted value ($\hat{T}$) and target (*T*) value for WT2 over the train dataset. (**f**) ANN predicted value ($\hat{T}$) and target (*T*) value for WT2 over the test dataset. (**g**) Absolute difference value between the prediction and estimation, $|T-\hat{T}|$, for WT2 over the train dataset. (**h**) Absolute difference value between the prediction and estimation, $|T-\hat{T}|$, for WT2 over the test dataset.

Figure 16a,b show the ANN predicted value ($\hat{T}$) and target (*T*) value for WT1 over the train and test dataset, respectively. Recall that this WT is healthy in both the training and test datasets. The prediction is close to the target in both figures, and only some samples on the test dataset have disparate values. This performance is shown in Figure 16c,d, where the absolute difference value between prediction and estimation, $|T-\hat{T}|$, also called residual, is shown for WT1 over the training and test datasets, respectively. This residual has similar values over the training and test sets, and only a few samples have peak values. As already mentioned in Section 5.6, if the residual, $|T-\hat{T}|$, was used directly to establish a threshold above which it is decided to give the alarm signal, this would lead to a non-assumable amount of false positives. Thus, the importance of defining an indicator, see Section 5.7, which considers the persistence of consecutive samples above a specified threshold.

In contrast, Figure 16e,f present the ANN predicted values ($\hat{T}$) and target (*T*) values for WT2 over the training and test datasets, respectively. Recall that this WT had a main bearing fault on 21 May 2018. These figures show that the prediction over the test set has an overall performance that is different from that over the training set. Note that Figure 16g,h, which represent the residuals on the training and test datasets, also contribute to observing this change in performance over the two sets. It is noteworthy that after the failure (on 21 May 2018), the residual is no longer affected and has a similar performance to the one on the train set, with only a few isolated peaks.

**Figure 17:**
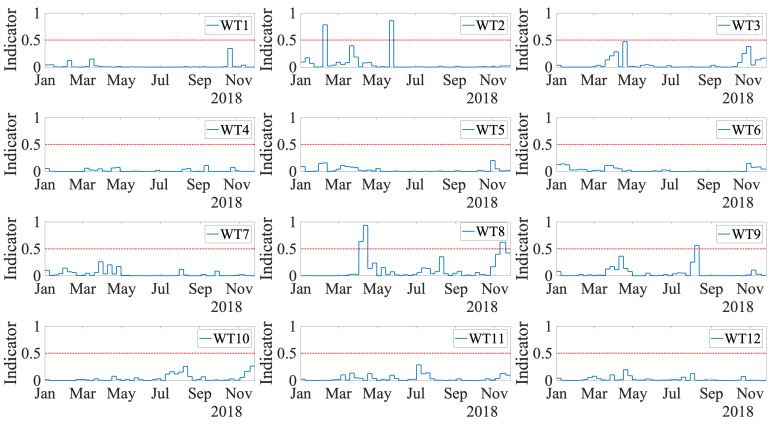
ANN indicator values (blue line) for test data, and threshold (red line).

Finally, the results obtained with the indicator proposed in Section 5.7 are shown in Figure 17 for the test dataset (2018) over the entire wind park. An alarm is triggered only when the indicator reaches a value greater than 0.5. The first WT with a triggered alarm was WT2, which is a true positive. The alarm was activated on 4 February; thus, three and a half months in advance of the actual breakdown reported on 21 May, where the low-speed shaft had to be replaced with a new one. The alarm stayed until 12 February and was then set off. This is because the possible heat created from an initial failure mode is detected by the fault prognosis methodology, but its appearance is not continuous over time until the final breakdown. In contrast, when the failure mode advances, for example, when a crack propagates, the generated heat appears. When the crack remains still, no further heat is generated; thus, the alarm is set off. However, cracks are already present and can advance at any time, leading to the possible failure of the component. Thus, in this methodology, whenever the alarm is on (even when it is set off after a few weeks), it is highly recommended to check the specific WT. The second WT with a triggered alarm was the WT8. This WT has no main bearing damage, but the work orders show that the gearbox was broken and replaced from 22 March to 11 April. The proposed indicator triggered an alarm on 1 April. Thus, the method detects this maintenance operation on the gearbox as an anomaly. In a real situation, the wind park manager knows that this WT is already under maintenance. Thus, it was not a false positive. The final WT with a triggered alarm was WT9. This is a false positive of the method, as this WT had no important work orders during the year 2018. The rest of the WTs in the park were correctly classified as healthy over all the test datasets. To summarize, the results lead to a precision of 50% and a recall of 100% in the wind farm under study.

Finally, the obtained results are compared to other approaches in the literature coping with the same proposed problem. The methodologies stated in References [15,17,23] achieved promising results for the diagnosis and prediction of WT faults from SCADA data. However, the obtained results in this present work surpass the ones given in the aforementioned references for two main reasons: (i) the prognosis is accomplished months in advance instead of only hours in advance of the fault, and (ii) as the proposed approach is unsupervised it does not need previous faulty data to be trained neither has to deal with the problem of the highly imbalanced nature of fault data (with the no-fault class having an overwhelming majority of samples).

## 7. Conclusions

In this work, an advanced prognostic system was proposed and proven to predict the main bearing failure before it occurs and let turbine operators plan their operations. In particular, a fault prognosis methodology that uses solely SCADA data requires only healthy data to be deployed. Furthermore, the stated strategy works under different operating and environmental conditions to which WTs are subject. Finally, the validity and performance of the established methodology were demonstrated on a real underproduction wind farm composed of 12 WTs. The results show that the time that the early prognosis can be generated is several months in advance, thus giving time to the plant operator to schedule maintenance. However, the studied wind farm has only one case with a failure of interest, which is not sufficient for statistical analysis. To set the expected predictive time and its confidence level is a future work that can be assessed when more cases appear in this wind farm, or data from other wind farms with more cases related to this fault are available. Furthermore, note that the work orders available to the authors of this work only contain those related to important systems substitutions, such as gearbox, generator, or main bearing replacement. Thus, preventive maintenance work orders, or minor work orders are not available. As future work, it would be interesting to check whether values of the indicator lower than the threshold but close to it are related to some minor work orders. Finally, other neural networks, such as long short-term memory (LSTM), recurrent neural network (RNN), or one dimension convolutional neural network (1D-CNN), should be studied, as they have interesting properties that, initially, make them appropriate for the problem under consideration.

## Figures and Tables

**Table 1:** Technical specifications of the WTs in the park.

Number of blades	3
Nominal power	1500 kW
Rotor diameter	77 m
Wind class	IEC IIa
Swept area	4657 m^2^
Nominal rotation speed	18.3 rpm
Cut-in wind speed	3.5 m/s
Cut-out wind speed	25 m/s
Bearings	Double spherical roller bearings
Power regulation	Independent pitch (variable speed)

**Table 2:** Environmental variables.

Variable	Description	Units
TempAmb	Ambient temperature	°C
TempGond	Nacelle temperature	°C
VelViento	Wind speed	m/s
IndTurbul	Turbulence index	-

**Table 3:** Electrical variables.

Variable	Description	Units
Pot	Active power	kW
TensRed	Phase voltage	V
CosPhi	Power factor	-
TotPotReact	Reactive power	kW
FrecRed	Electric network frequency	Hz

**Table 4:** Component temperature variables.

Variable	Description	Units
TempAceiteGH	Hydraulic group oil temperature	°C
TempAceiteMultip	Gearbox oil temperature	°C
TempRodamMultip	Gearbox bearing temperature	°C
TempGen	Generator temperature	°C
TempRodamTrasero	Rear bearing temperature	°C
TempCojLA	Bearing coupling side temperature	°C
TempCojLOA	Bearing non-coupling side temperature	°C
TempRadSup	Upper gearbox radiator temperature	°C
TempRadInf	Lower gearbox radiator temperature	°C
TempEjeLento_1	Low-speed shaft temperature	°C
TempTrafo1	Transformer 1 temperature	°C
TempTrafo2	Transformer 2 temperature	°C
TempTrafo3	Transformer 3 temperature	°C

**Table 5:** Hydraulic variables.

Variable	Description	Units
AcumGralPala1	General accumulator blade 1	bar
AcumGralPala2	General accumulator blade 2	bar
AcumGralPala3	General accumulator blade 3	bar
PresAcumGral	Accumulated general pressure	bar
PresFreno	Brake pressure	bar
PresGH	Hydraulic group pressure	bar

**Table 6:** Control variables.

Variable	Description	Units
Pitch1	Blade pitch angle 1	°C
Pitch2	Blade pitch angle 2	°C
Pitch3	Blade pitch angle 3	°C
Yaw	Yaw angle	°C
VelRotor	Rotor speed	rpm
VelGen	Generator speed	rpm
SPPitch	Pitch system parameter	-
ContEnerActiva	Active energy counter	-
NivOscil	Oscillation level	Hz
NivVibra	Vibration level	Hz
date_time	Date and time of the sample	-
ld_id	Wind turbine ID	-

**Table 7:** Selected SCADA variables used to develop the normality model, its description, range of possible values, and units. All of them are related to the mean value over a 10-min period.

Variable	Description	Range	Units
Pot	Generated real power	[0,2000]	kW
TempAmb	ambient temperature	[−5,40]	°C
TempCojLA	Bearing coupling side temperature	[0,120]	°C
TempCojLOA	Bearing non-coupling side temperature	[0,120]	°C
TempEjeLento	Low-speed shaft temperature	[0,120]	°C
TempGen	generator temperature	[0,175]	°C
TempRodamMultip	Gearbox temperature	[0,120]	°C
VelRotor	Rotor speed	[0,50]	rpm

**Table 8:** Mean squared error (MSE) of the trained models for each WT. In all cases, the training time is close to 25 min (±30 s) on a 3.2 GHz 6-Core Intel Core i7 processor.

WT	Train Error (MSE)
WT1	0.6984
WT2	1.3104
WT3	6.1074
WT4	0.7227
WT5	5.9989
WT6	5.2275
WT7	0.9214
WT8	1.8503
WT9	0.7373
WT10	3.8815
WT11	4.6074
WT12	4.0173

## Data Availability

The data required to reproduce these findings cannot be shared at this time as it is proprietary.
